# Effectiveness, safety, and healthcare costs associated with rivaroxaban versus warfarin among venous thromboembolism patients with obesity: a real-world study in the United States

**DOI:** 10.1007/s11239-022-02661-1

**Published:** 2022-05-13

**Authors:** Jeffrey S. Berger, François Laliberté, Akshay Kharat, Dominique Lejeune, Kenneth Todd Moore, Young Jung, Patrick Lefebvre, Veronica Ashton

**Affiliations:** 1 New York University Langone Health, New York, NY USA; 2 Groupe d’analyse, Ltée, 1190 avenue des Canadiens-de-Montréal, Suite 1500, Montréal, QC H3B 0G7 Canada; 3 Janssen Scientific Affairs, LLC., Titusville, NJ USA; 4 Janssen Pharmaceuticals, Inc., Titusville, NJ USA

**Keywords:** Venous thromboembolism, Rivaroxaban, Recurrence, Major bleeding, Healthcare costs, Real world

## Abstract

Prior observational studies suggest rivaroxaban is safe and effective among patients with morbid obesity who suffered a venous thromboembolism (VTE) event, but existing data are more limited in the broader population of VTE patients with obesity. This study assessed VTE recurrence, major bleeding, healthcare resource utilization, and healthcare costs among VTE patients with obesity who received rivaroxaban versus warfarin. VTE patients with obesity who initiated rivaroxaban or warfarin after a first VTE (index date) were identified from the IQVIA PharMetrics® Plus database (01/02/2011–09/30/2019). The follow-up period spanned from the index date until health plan disenrollment, end of data availability, cancer diagnosis/treatment, end of the 12 month post-index period, or (for the analysis of major bleeding) anticoagulant discontinuation or switch. Patient characteristics were balanced using inverse probability of treatment weighting. The weighted rivaroxaban (N = 8666) and warfarin cohorts (N = 5946) were well balanced (mean age = 51 years, females = 52%). Over a 9.6 months mean observation period, rivaroxaban users had a significantly lower risk of VTE recurrence [7.0% vs. 8.2%, HR(95% CI) = 0.85(0.75;0.97)] and a similar risk of major bleeding [4.1% vs. 3.6%, HR(95% CI) = 1.11(0.89;1.37)] relative to warfarin users at 12 months. Relative to warfarin users, rivaroxaban users had significantly fewer all-cause outpatient visits [RR(95% CI) = 0.71(0.70;0.74)]. The higher pharmacy costs incurred by rivaroxaban recipients (cost difference = $1252) were offset by lower medical costs (cost difference = − $2515, all p < 0.05) compared with warfarin recipients. Our findings suggest that rivaroxaban is safe and effective versus warfarin, and associated with lower medical costs among VTE patients with obesity.

**Supplementary Information:**

The online version contains supplementary material available at 10.1007/s11239-022-02661-1.

## Highlights

- Data on the use of rivaroxaban in VTE patients with obesity are limited
- In this study, rivaroxaban was associated with fewer VTE recurrences than warfarin
- Rivaroxaban-initiated patients had similar rates of major bleeding vs. warfarin
- Higher pharmacy costs with rivaroxaban were fully offset by medical cost savings
- Rivaroxaban is a safe and effective treatment vs. warfarin for VTE obese patients

## Introduction

Obesity is a serious public health issue that affects a growing number of individuals in the United States (US) [1]. In 2008, US medical costs associated with obesity were estimated at $147 billion [2]. The condition is associated with a chronic hypercoagulable state that increases the risk of venous thromboembolism (VTE) by at least two fold [3, 4].

Direct acting oral anticoagulants (DOACs) are the standard treatment to prevent VTE recurrence in patients who previously experienced a VTE event [5]. Until recently, data on their use in obese population were more limited, and current labeling information does not recommend any dose adjustments in VTE patients with obesity. Mounting evidence from observational studies suggests DOACs may be a safe and effective alternative to vitamin K antagonists (VKAs; such as warfarin) in patients with morbid obesity (i.e., BMI ≥ 40 kg/m^2^) [6–11], and some studies further support that they may have a similar profile in the broader population of patients with overall obesity (i.e., BMI ≥ 30 kg/m^2^) [12–16]. Rivaroxaban may be particularly effective in VTE patients with morbid obesity [11]. However, data on the comparative safety and effectiveness of rivaroxaban versus warfarin as a VTE treatment are limited in the broader population of patients with overall obesity. Furthermore, healthcare costs associated with the use of rivaroxaban and warfarin remain uncertain in this population. To fill this knowledge gap, the current study sought to assess VTE recurrence, major bleeding, healthcare resource utilization (HRU), and healthcare costs among patients with obesity who had an acute VTE event and received treatment with rivaroxaban or warfarin.

## Materials and methods

### Data source

Patients were identified from IQVIA PharMetrics® Plus database (study period: 11/02/2011–09/30/2019; IQVIA database). This database comprises data on enrollees and is representative across US regions, with medical and pharmacy benefits available in any given recent year. Information on ~ 40 million patients is available and is generally representative of the less-than-65 years of age, commercially-insured population with respect to both age and sex. The IQVIA database contains information on demographics; plan enrollment; and inpatient, outpatient, and pharmacy claims and associated costs. Available data are fully de-identified and therefore compliant with the Health Insurance Portability and Accountability Act.

### Study design and study population

A retrospective, observational cohort study was conducted. Index date was defined as the date of initiation of rivaroxaban or warfarin ≤ 30 days after a first VTE event [i.e., ≥ 1 medical claim with VTE diagnosis in any position (Table S1 and S2 for codes)]. The baseline period was defined as the 12-month period before the index date; patient characteristics were evaluated during this period. Patients with an index date on or after 01/01/2014 were included to account for potential differences associated with early adopters of rivaroxaban (approved 11/2012 by the Food and Drug Administration) and delays before the wider use of the drug. Patients were additionally required to have: (1) ≥ 12 months of continuous health plan enrollment pre-index date, (2) ≥ 1 medical claim with a diagnosis of obesity/BMI ≥ 30 kg/m^2^ (Table S3 for BMI-related International Classification of Diseases [ICD] codes) during the baseline period or on index date, and (3) ≥ 18 years old at index date.

The following exclusion criteria were applied: (1) presence of claims for multiple oral anticoagulants on index date, (2) ≥ 1 pharmacy claim for an oral anticoagulant during the baseline period, (3) ≥ 1 medical claim for VTE before the first VTE event during the baseline period, (4) recurrent VTE after the first observed VTE event but prior to the index date, (5) knee or hip replacement surgery during the baseline period, (6) ≥ 1 medical claim with a diagnosis of atrial fibrillation during the baseline period, (7) cancer diagnosis and treatment during the baseline period or on the index date.

Effectiveness, HRU, and costs were assessed using an intention-to-treat (ITT) approach, whereas safety was assessed using an on-treatment approach. The ITT follow-up spanned from the index date until health plan disenrollment, end of data availability, presence of both cancer diagnosis and treatment (at the later of the two dates), or 12 months, whichever came first. The on-treatment follow-up was censored similarly to the ITT approach and additionally upon anticoagulant discontinuation or anticoagulant switch, so patients were continuously treated with the index anticoagulant. Treatment discontinuation was defined as a gap of ≥ 60 days of supply between the end of an anticoagulant dispensing and the next medication refill or end of data availability. Effectiveness was also assessed using an on-treatment approach in a sensitivity analysis.

### Study outcomes

The effectiveness outcome was VTE recurrence, defined as a hospitalization with a primary diagnosis of VTE. Major bleeding was the safety outcome and was identified using hospitalizations with indicators (diagnoses and procedures) of a bleeding episode based on the Cunningham algorithm [17].

All-cause and VTE-related HRU and healthcare costs were assessed during follow-up. HRU outcomes included hospitalizations and days of hospital stay, emergency room (ER) visits, and outpatient visits. Outpatient visits were further broken down into office, outpatient hospital, and other outpatient (including patient home and other unlisted facilities). All-cause healthcare costs included medical and pharmacy costs, with medical costs further broken down into the same categories as HRU. VTE-related HRU and costs were defined as visits/costs with primary or secondary diagnosis (i.e., identified in any other diagnosis fields) of VTE [18].

### Statistical analysis

Inverse probability of treatment weighting (IPTW) was used to balance the characteristics of cohorts. IPTW uses weights derived from the propensity score (PS) to create pseudo-populations, so that covariates are distributed independently of treatment assignment. PS was defined as the conditional probability of receiving rivaroxaban based on observable covariates. The following covariates were included in the PS estimation: age, sex, year of index date, region, type of insurance plan, morbid obesity (i.e., BMI ≥ 40 kg/m^2^), time between VTE event and index date, type of VTE event, baseline major bleeding, cardiovascular-related medications, cardiovascular procedures, use of non-oral anticoagulants, number of unique prescription drugs used during baseline, baseline healthcare resource utilization and costs, and baseline risk factors for VTE and bleeding events (with ≥ 1% prevalence in either cohort). Weights were truncated at the 99% of the distribution to limit the effect of extreme weights. The balancing of patient baseline characteristics was assessed using standardized differences, with a threshold < 10% considered not clinically meaningful [19].

Weighted Kaplan–Meier (KM) survival analysis was used to assess time to VTE recurrence and time to major bleeding events. Cumulative KM rates were reported at 12 months post-index date. Rates of VTE recurrence and major bleeding were compared between cohorts using weighted Cox proportional hazards regression models; corresponding hazard ratios (HR), 95% confidence intervals (CI), and p-values were reported.

HRU and healthcare costs were evaluated per patient-years (PPY) to account for the variable duration of follow-up among individual patients. Rates of HRU were compared between cohorts using rate ratios (RR) obtained from Poisson regression models. Costs were compared between cohorts using mean cost differences. Costs were inflated to 2019 US dollars using the medical care component of the Consumer Price Index. Non-parametric bootstrap procedures were used to estimate 95%CI and p-values for comparisons, since HRU and cost data have positive values that follow a non-normal distribution and commonly include zero values.

## Results

### Baseline characteristics

After applying all study selection criteria, 8666 patients were included in the rivaroxaban cohort and 5946 were included in the warfarin cohort. Patient baseline characteristics were adequately balanced by IPTW (Table 1, Figure S1). After weighting, mean age was 51 years in both cohorts. The rivaroxaban and warfarin cohorts comprised 51.8% and 51.6% of female patients, respectively. The type of VTE experienced at baseline, including pulmonary embolism (PE; rivaroxaban:28.7%, warfarin:29.6%), deep vein thrombosis (DVT; rivaroxaban:50.5%, warfarin:49.2%), or both (rivaroxaban:20.8%, warfarin:21.3%), was also similar between cohorts. All-cause and VTE-related HRU were well balanced at baseline, and average all-cause total healthcare costs were similar between the rivaroxaban ($47,814 PPY) and warfarin cohorts ($49,123 PPY).

**Table 1:** Baseline demographics and clinical characteristics of the vte patients with obesity treated with rivaroxaban or warfarin

	Unweighted cohorts			Weighted cohorts^a^		
	Rivaroxaban	Warfarin	Std. diff.^b,c^	Rivaroxaban	Warfarin	Std. diff.^b,c^
	N = 8666	N = 5946	(%)	N = 8666	N = 5946	(%)
Demographics^d^						
Age, years, mean ± SD [median]	50.9 ± 11.6 [53]	51.6 ± 11.7 [53]	6.1	51.1 ± 11.6 [53]	51.3 ± 11.6 [53]	1.5
Sex, female, n (%)	4378 (50.5)	3102 (52.2)	3.3	4489 (51.8)	3067 (51.6)	0.4
Year of index date,^d^ n (%)						
2014	1227 (14.2)	1752 (29.5)	37.1	1817 (21.0)	1318 (22.2)	2.9
2015	1427 (16.5)	1455 (24.5)	19.8	1726 (19.9)	1243 (20.9)	2.5
2016	1633 (18.8)	1008 (17.0)	4.9	1585 (18.3)	1110 (18.7)	1.0
2017	1700 (19.6)	773 (13.0)	17.9	1441 (16.6)	955 (16.1)	1.5
2018	1623 (18.7)	602 (10.1)	24.5	1290 (14.9)	836 (14.1)	2.3
2019	1056 (12.2)	356 (6.0)	21.6	807 (9.3)	484 (8.1)	4.2
Region,^d^ n (%)						
South	1872 (21.6)	1488 (25.0)	8.1	1950 (22.5)	1349 (22.7)	0.4
Midwest	2403 (27.7)	1938 (32.6)	10.6	2600 (30.0)	1825 (30.7)	1.5
Northeast	3729 (43.0)	1842 (31.0)	25.0	3291 (38.0)	2196 (36.9)	2.1
West	662 (7.6)	678 (11.4)	12.8	825 (9.5)	576 (9.7)	0.6
Insurance plan type,^d^ n (%)						
PPO	7613 (87.8)	5067 (85.2)	7.7	7521 (86.8)	5145 (86.5)	0.7
HMO	566 (6.5)	461 (7.8)	4.7	618 (7.1)	432 (7.3)	0.5
POS	347 (4.0)	225 (3.8)	1.1	340 (3.9)	212 (3.6)	1.8
Indemnity/traditional	105 (1.2)	156 (2.6)	10.3	142 (1.6)	125 (2.1)	3.5
Unknown	27 (0.3)	32 (0.5)	3.5	37 (0.4)	27 (0.5)	0.5
CDHC	8 (0.1)	5 (0.1)	0.3	9 (0.1)	4 (0.1)	1.1
Time between VTE event date and index date,^e^ days, mean ± SD [median]	4.5 ± 7.9 [1]	2.5 ± 5.7 [0]	28.7	3.6 ± 7.1 [0]	3.1 ± 6.5 [0]	7.4
VTE event type,^e^ n (%)						
PE	2325 (26.8)	1802 (30.3)	7.7	2483 (28.7)	1758 (29.6)	2.0
DVT	4754 (54.9)	2652 (44.6)	20.5	4379 (50.5)	2923 (49.2)	2.7
PE and DVT	1587 (18.3)	1492 (25.1)	16.4	1803 (20.8)	1264 (21.3)	1.1
Baseline medication,^f^ n (%)						
Dispensing of unique prescription drugs,^g^ mean ± SD [median]	12.8 ± 10.4 [10]	13.2 ± 10.7 [11]	3.7	13.1 ± 10.6 [11]	13.1 ± 10.6 [11]	0.6
Non-oral anticoagulants,^h^ n (%)	1742 (20.1)	1539 (25.9)	13.7	2000 (23.1)	1427 (24.0)	2.2
Cardiovascular-related medications, n (%)						
Antihyperlipidemic agents	2509 (29.0)	1875 (31.5)	5.6	2586 (29.8)	1779 (29.9)	0.2
Antihypertensive agents	3952 (45.6)	2962 (49.8)	8.4	4113 (47.5)	2839 (47.7)	0.6
Antiplatelet agents	257 (3.0)	224 (3.8)	4.4	292 (3.4)	205 (3.4)	0.4
Gastric bypass surgery,^f^ n (%)	107 (1.2)	136 (2.3)	8.0	138 (1.6)	117 (2.0)	2.8
Cardiovascular procedures,^f^ n (%)	208 (2.4)	272 (4.6)	11.9	280 (3.2)	200 (3.4)	0.7
Coronary bypass graft	54 (0.6)	86 (1.4)	8.1	71 (0.8)	64 (1.1)	2.5
Percutaneous coronary intervention	164 (1.9)	204 (3.4)	9.6	222 (2.6)	149 (2.5)	0.4
Baseline healthcare resource utilization,^f^ mean ± SD [median]						
All-cause						
Hospitalizations	0.8 ± 1.0 [1]	1.7 ± 0.9 [1]	39.5	1.0 ± 1.0 [1]	1.0 ± 0.9 [1]	3.5
ER visits	1.3 ± 2.3 [1]	1.1 ± 2.2 [1]	7.9	1.2 ± 2.2 [1]	1.2 ± 2.2 [1]	1.2
OP visits	19.0 ± 18.2 [14]	20.7 ± 24.2 [15]	8.0	19.6 ± 20.5 [14]	19.8 ± 21.2 [14]	1.1
OP hospital visits	4.2 ± 6.5 [2]	5.3 ± 11.7 [2]	11.7	4.5 ± 7.5 [2]	4.7 ± 9.3 [2]	2.5
Office visits	10.5 ± 11.5 [7]	9.9 ± 11.2 [7]	5.4	10.2 ± 11.3 [7]	10.2 ± 11.5 [7]	0.6
Other visits	4.3 ± 8.2 [2]	5.5 ± 14.1 [2]	10.4	4.9 ± 11.4 [2]	5.0 ± 11.4 [2]	0.8
VTE-related^i^						
Hospitalizations	0.5 ± 0.5 [0]	0.8 ± 0.5 [1]	58.2	0.6 ± 0.5 [1]	0.6 ± 0.5 [1]	4.9
ER visits	0.4 ± 0.5 [0]	0.2 ± 0.4 [0]	39.6	0.3 ± 0.5 [0]	0.3 ± 0.5 [0]	4.8
OP visits	0.5 ± 0.9 [0]	0.4 ± 1.2 [0]	15.3	0.4 ± 1.0 [0]	0.4 ± 1.0 [0]	1.8
OP hospital visits	0.2 ± 0.5 [0]	0.2 ± 0.6 [0]	14.0	0.2 ± 0.5 [0]	0.2 ± 0.5 [0]	0.8
Office visits	0.2 ± 0.5 [0]	0.1 ± 0.3 [0]	31.1	0.2 ± 0.4 [0]	0.1 ± 0.4 [0]	5.9
Other visits	0.1 ± 0.5 [0]	0.1 ± 0.9 [0]	5.6	0.1 ± 0.7 [0]	0.1 ± 0.7 [0]	1.6
Baseline healthcare costs,^f^ $US 2019, mean ± SD						
All-cause						
Total healthcare costs	$36,405 ± 60,061	$61,844 ± 94,492	32.1	$47,814 ± 79,717	$49,123 ± 80,041	1.6
Total medical costs	$33,148 ± 57,949	$58,406 ± 92,537	32.7	$44,493 ± 78,089	$45,715 ± 77,171	1.6
Hospitalization costs	$22,326 ± 52,904	$45,988 ± 84,920	33.4	$33,313 ± 73,442	$34,123 ± 70,023	1.1
ER costs	$2243 ± 5272	$1978 ± 5475	4.9	$2106 ± 5131	$2088 ± 5285	0.3
OP costs	$8579 ± 16,249	$10,440 ± 29,253	7.9	$9074 ± 17,464	$9504 ± 24,974	2.0
OP hospital visit costs	$5521 ± 12,793	$6541 ± 23,045	5.5	$5845 ± 13,645	$5903 ± 18,410	0.4
Office visit costs	$1435 ± 2473	$1479 ± 2770	1.7	$1434 ± 2463	$1482 ± 2723	1.8
Other visit costs	$1623 ± 7264	$2420 ± 15,074	6.7	$1795 ± 7982	$2119 ± 14,486	2.8
Pharmacy costs	$3257 ± 10,503	$3438 ± 12,667	1.6	$3321 ± 10,133	$3408 ± 15,166	0.7
VTE-related^i^						
Total healthcare costs	$14,309 ± 38,453	$33,575 ± 72,389	33.2	$23,272 ± 60,114	$23,809 ± 57,093	0.9
Hospitalization costs	$13,121 ± 38,605	$32,938 ± 72,562	34.1	$22,324 ± 60,301	$22,933 ± 57,323	1.0
ER costs	$815 ± 2268	$424 ± 1854	18.9	$638 ± 2158	$625 ± 2047	0.6
OP costs	$374 ± 2144	$213 ± 1525	8.6	$311 ± 1926	$251 ± 1386	3.6
OP hospital visit costs	$291 ± 1800	$139 ± 899	10.7	$238 ± 1601	$172 ± 970	5.0
Office visit costs	$51 ± 783	$22 ± 133	5.2	$39 ± 733	$36 ± 177	0.6
Other visit costs	$32 ± 814	$52 ± 1,212	1.9	$33 ± 731	$43 ± 966	1.2

*CDHC* community driven healthcare, *DVT* deep vein thrombosis, *ER* emergency room, *HMO* health maintenance organization, *OP* outpatient, *PE* pulmonary embolism, *POS* point of service, *PPO* preferred provider organization, *SD* standard deviation, *Std. diff* standardized difference, *VTE* venous thromboembolism

^a^Rivaroxaban and warfarin patients were weighted using the inverse probability of treatment weighting approach based on the propensity score

^b^For continuous variables, the standardized difference is calculated by dividing the absolute difference in means of the control and the case by the pooled standard deviation of both groups. The pooled standard deviation is the square root of the average of the squared standard deviations

^c^For dichotomous variables, the standardized difference is calculated using the following equation where P is the respective proportion of participants in each group: |(P_case_-P_control_)|/√[(P_case_(1-P_case_) + P_control_(1-P_control_))/2]

^d^Evaluated at the index date

^e^Defined as a primary or secondary diagnosis of PE or DVT

^f^Evaluated during the 12 months prior to the index date, excluding the index date

^g^Prescription drugs were based on unique National Drug Codes

^h^Includes unfractionated heparin, fondaparinux, and low molecular weight heparin

^i^HRU and healthcare costs are considered VTE-related if it is associated with a primary or secondary diagnosis of venous thromboembolism

The proportion of VTE patients with morbid obesity (i.e., BMI ≥ 40 kg/m^2^) was 41.1% in the rivaroxaban cohort and 41.9% in the warfarin cohort (Table 2). On average, the Quan-Charlson comorbidity index was 1.2 in the rivaroxaban cohort and 1.3 in the warfarin cohort. The most prevalent risk factors for VTE and bleeding events were hypertension (rivaroxaban:63.3%, warfarin:63.8%) and diabetes (rivaroxaban:28.0%, warfarin:28.5%).

**Table 2:** Baseline Risk Factors of the VTE Patients with Obesity Treated with Rivaroxaban or Warfarin

	Unweighted cohorts			Weighted cohorts^a^		
	Rivaroxaban	Warfarin	Std. diff.^b,c^	Rivaroxaban	Warfarin	Std. diff.^b,c^
	N = 8666	N = 5946	(%)	N = 8666	N = 5946	(%)
Morbid obesity^d^ (BMI ≥ 40), n (%)	3157 (36.4)	2736 (46.0)	19.5	3565 (41.1)	2493 (41.9)	1.6
Quan-CCI,^e^ mean ± SD [median]	1.0 ± 1.5 [0]	1.5 ± 1.9 [1]	27.4	1.2 ± 1.7 [1]	1.3 ± 1.7 [1]	5.0
RIETE,^e^ mean ± SD [median]	1.3 ± 1.3 [1]	1.8 ± 1.5 [1]	34.5	1.5 ± 1.4 [1]	1.5 ± 1.5 [1]	5.1
Baseline major bleeding,^e,f^ n (%)	268 (3.1)	429 (7.2)	18.6	395 (4.6)	319 (5.4)	3.7
Baseline comorbidities,^e^ n (%)						
VTE and bleeding risk factors						
Hypertension	5259 (60.7)	3975 (66.9)	12.8	5486 (63.3)	3792 (63.8)	1.0
Diabetes	2182 (25.2)	1885 (31.7)	14.5	2423 (28.0)	1694 (28.5)	1.2
Arrhythmia (excluding AF)	1059 (12.2)	1167 (19.6)	20.2	1308 (15.1)	936 (15.7)	1.8
Myocardial infarction	457 (5.3)	506 (8.5)	12.8	567 (6.5)	403 (6.8)	1.0
Prior stroke	206 (2.4)	360 (6.1)	18.3	331 (3.8)	249 (4.2)	1.9
Other VTE risk factors						
Hyperlipidemia	4036 (46.6)	2907 (48.9)	4.6	4118 (47.5)	2838 (47.7)	0.4
Multiple trauma	3245 (37.4)	2242 (37.7)	0.5	3252 (37.5)	2228 (37.5)	0.1
Other serious infections	2203 (25.4)	1830 (30.8)	11.9	2408 (27.8)	1675 (28.2)	0.8
Major surgery	2137 (24.7)	1983 (33.4)	19.2	2477 (28.6)	1737 (29.2)	1.4
Abdomen surgery	1447 (16.7)	1444 (24.3)	18.8	1753 (20.2)	1223 (20.6)	0.8
CAD	1099 (12.7)	1063 (17.9)	14.4	1292 (14.9)	904 (15.2)	0.9
Pneumonia	1096 (12.6)	1013 (17.0)	12.3	1276 (14.7)	893 (15.0)	0.8
Contraceptive pill (use of oral)	817 (9.4)	474 (8.0)	5.2	753 (8.7)	504 (8.5)	0.7
Congestive heart failure	764 (8.8)	879 (14.8)	18.5	981 (11.3)	704 (11.8)	1.6
Hip, pelvis or leg fracture	601 (6.9)	388 (6.5)	1.6	597 (6.9)	400 (6.7)	0.6
COPD	590 (6.8)	456 (7.7)	3.3	635 (7.3)	440 (7.4)	0.3
PAD	419 (4.8)	418 (7.0)	9.3	482 (5.6)	357 (6.0)	1.9
Thrombocytopenia (low platelet count)	315 (3.6)	383 (6.4)	12.8	412 (4.8)	298 (5.0)	1.2
Varicose veins	294 (3.4)	254 (4.3)	4.6	340 (3.9)	235 (4.0)	0.2
Surgical resection of abdominal or pelvic cancer	192 (2.2)	182 (3.1)	5.3	243 (2.8)	164 (2.8)	0.3
Rheumatoid arthritis	176 (2.0)	152 (2.6)	3.5	192 (2.2)	135 (2.3)	0.4
Pregnancy	163 (1.9)	169 (2.8)	6.3	211 (2.4)	144 (2.4)	0.1
Inflammatory bowel disease	126 (1.5)	132 (2.2)	5.7	149 (1.7)	103 (1.7)	0.1
Spinal cord injury	71 (0.8)	55 (0.9)	1.1	80 (0.9)	50 (0.8)	0.8
Treatment with aromatase inhibitors	31 (0.4)	17 (0.3)	1.3	28 (0.3)	19 (0.3)	0.1
Immobility	29 (0.3)	28 (0.5)	2.2	31 (0.4)	25 (0.4)	0.9
Treatment with SERMs	10 (0.1)	8 (0.1)	0.5	8 (0.1)	8 (0.1)	0.9
Treatment with erythropoiesis stimulating agents	0 (0.0)	36 (0.6)	11.0	0 (0.0)	23 (0.4)	8.8
Other bleeding risk factors						
NSAID use	3159 (36.5)	1961 (33.0)	7.3	3031 (35.0)	2107 (35.4)	1.0
Excessive fall risk (Parkinson's disease, etc.)	2129 (24.6)	1534 (25.8)	2.8	2204 (25.4)	1522 (25.6)	0.4
Anemia	1687 (19.5)	1789 (30.1)	24.6	2081 (24.0)	1477 (24.8)	1.9
Renal disease	1687 (19.5)	1746 (29.4)	23.0	2039 (23.5)	1462 (24.6)	2.5
Ethanol abuse	1204 (13.9)	664 (11.2)	8.2	1102 (12.7)	747 (12.6)	0.5
Chronic kidney disease	1090 (12.6)	1116 (18.8)	17.0	1306 (15.1)	911 (15.3)	0.7
Previous bleeding	1090 (12.6)	1189 (20.0)	20.1	1351 (15.6)	958 (16.1)	1.4
Hepatic disease	970 (11.2)	859 (14.4)	9.7	1103 (12.7)	761 (12.8)	0.2
Central venous catheter	381 (4.4)	581 (9.8)	20.9	579 (6.7)	409 (6.9)	0.8
Left ventricular dysfunction	205 (2.4)	235 (4.0)	9.1	262 (3.0)	184 (3.1)	0.4
Coagulation defect	186 (2.1)	236 (4.0)	10.6	258 (3.0)	174 (2.9)	0.3
Thrombophilia	129 (1.5)	163 (2.7)	8.7	181 (2.1)	121 (2.0)	0.4
Peptic ulcer	124 (1.4)	170 (2.9)	9.9	177 (2.0)	125 (2.1)	0.4
Transient ischemic attack	99 (1.1)	104 (1.7)	5.1	120 (1.4)	92 (1.5)	1.3
Diathesis	3 (0.0)	1 (0.0)	1.1	3 (0.0)	1 (0.0)	1.3

*AF* atrial fibrillation, *BMI* body mass index, *CAD* coronary artery disease, *COPD* chronic obstructive pulmonary disease, *DVT* deep vein thrombosis; *NSAID* nonsteroidal anti-inflammatory drugs, *PAD* peripheral artery disease, *Quan-CCI* Quan-Charlson comorbidity index, *RIETE* registro informatizado enfermedad tromboembolica, *SERM* selective estrogen receptor modulator, *Std. diff* standard difference, *VTE* venous thromboembolism

^a^Rivaroxaban and warfarin patients were weighted using the inverse probability of treatment weighting approach based on the propensity score

^b^For continuous variables, the standardized difference is calculated by dividing the absolute difference in means of the control and the case by the pooled standard deviation of both groups. The pooled standard deviation is the square root of the average of the squared standard deviations

^c^For dichotomous variables, the standardized difference is calculated using the following equation where P is the respective proportion of participants in each group: |(P_case_-P_control_)| / √[(P_case_(1-P_case_) + P_control_(1-P_control_))/2]

^d^Evaluated at the closest date to the index date with a diagnosis of obesity during the 12 months prior to the index date, including the index date

^e^Evaluated during the 12 months prior to the index date, excluding the index date

^f^Major bleeding was identified with the Cunningham algorithm, which identifies hospitalizations with diagnoses and procedures indicating an episode of bleeding (excluding bleeding due to major trauma)

### Recurrence of venous thromboembolism and major bleeding

**Fig. 1:**
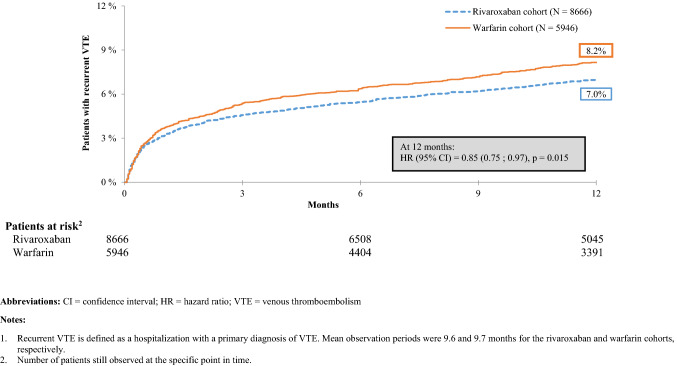
Kaplan–Meier Rates of Recurrent VTE^1^—Intention-to-Treat

**Fig. 2:**
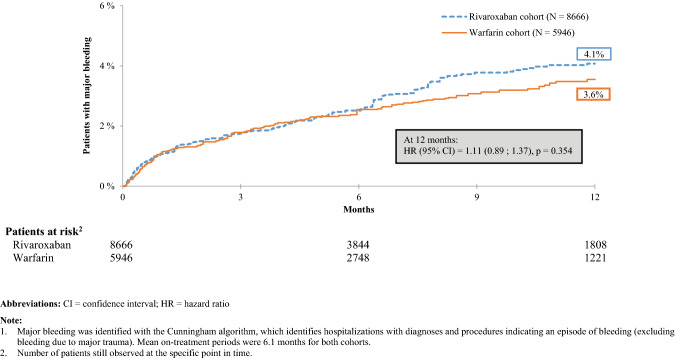
Kaplan–Meier Rates of Major Bleeding^1^—On-Treatment

Based on an ITT approach, patients in the rivaroxaban cohort had a significantly lower risk of VTE recurrence than those in the warfarin cohort at 12 months [HR(95% CI) = 0.85(0.75;0.97), p = 0.015; Fig. 1]. A similar effect was observed in the sensitivity analysis conducted using an on-treatment approach [HR(95%CI) = 0.86(0.75;0.99), p = 0.035]. There was no significant difference in the risk of major bleeding between groups at 12 months [HR(95%CI) = 1.11(0.89;1.37), p = 0.354; Fig. 2].

### Healthcare resource utilization and costs

The rates of all-cause hospitalization [RR(95%CI) = 0.94(0.86;1.06), p = 0.365] and ER visits [RR(95%CI) = 0.93(0.87;1.01), p = 0.108] were not significantly different between the rivaroxaban and warfarin cohorts (Table 3). Relative to patients in the warfarin cohort, those in the rivaroxaban cohort had significantly lower rates of outpatient visits [RR(95%CI) = 0.71(0.70;0.74)], including outpatient hospital visits [RR(95%CI) = 0.55(0.54;0.61)], office visits [RR(95%CI) = 0.92(0.89;0.96)], and other visits [RR(95%CI) = 0.62(0.59;0.67), all p < 0.001]. Similar results were generally observed when assessing VTE-related HRU, with the exception that patients in the rivaroxaban cohort exhibited significantly lower rates of VTE-related ER visits than those in the warfarin cohort [RR(95%CI) = 0.76(0.67;0.87), p < 0.001].

**Table 3:** Healthcare Resource Utilization among Rivaroxaban vs. Warfarin Cohorts up to 12 months post-index date – Intention-to-Treat

Healthcare resource utilization	Rate (per patient-years)		Rate ratio^a,b^ (95% CI) [A]/[B]	P-value^a,b^
	Rivaroxaban	Warfarin		
	[A]	[B]		
Observation period,^c^ months, mean ± SD [median]	9.7 ± 3.8 [12]	9.6 ± 3.8 [12]		
Total patient-years	6896	4703		
All-cause				
Hospitalizations	0.4	0.4	0.94 (0.86, 1.06)	0.365
LOS, days, mean [median]	8.2 [5]	8.6 [5]		
ER visits	1.1	1.2	0.93 (0.87, 1.01)	0.108
OP visits	29.7	41.8	0.71 (0.70, 0.74)	< 0.001
OP hospital visits	7.4	13.4	0.55 (0.54, 0.61)	< 0.001
Office visits	14.4	15.8	0.92 (0.89, 0.96)	< 0.001
Other visits	7.9	12.7	0.62 (0.59, 0.67)	< 0.001
VTE-related^d^				
Hospitalizations	0.2	0.2	0.91 (0.80, 1.05)	0.228
LOS, days, mean [median]	9.4 [5]	9.8 [5]		
ER visits	0.2	0.2	0.76 (0.67, 0.87)	< 0.001
OP visits	5.5	13.7	0.40 (0.39, 0.42)	< 0.001
OP hospital visits	1.6	5.4	0.29 (0.27, 0.31)	< 0.001
Office visits	2.9	4.6	0.64 (0.61, 0.67)	< 0.001
Other visits	1.0	3.7	0.27 (0.25, 0.31)	< 0.001

*CI* confidence interval, *ER* emergency room, *LOS* length of stay, *OP* outpatient, *VTE* venous thromboembolism

^a^Rate ratios obtained from Poisson regression models

^b^Confidence intervals and p-values were calculated using non-parametric bootstrap procedure (B = 499)

^c^The observation period spans from the index date until the earliest date between 12 months, health plan disenrollment, end of data availability, or presence of both diagnosis and treatment of cancer (at the later of the two dates)

^d^HRU is considered VTE-related if it is associated with a primary or secondary diagnosis (i.e., identified in any other diagnosis fields) of venous thromboembolism

**Fig. 3:**
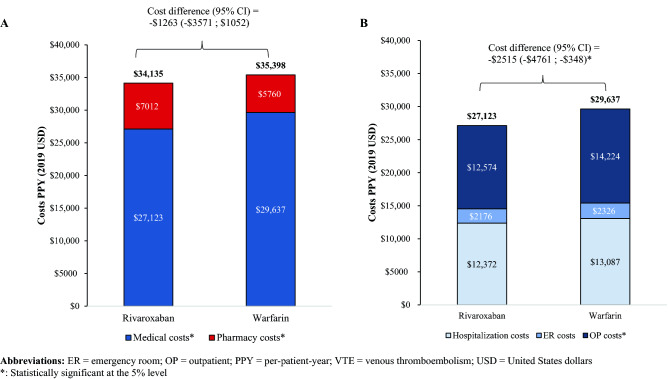
**A** Total All-Cause Healthcare Costs, Stratified into Medical and Pharmacy Costs, and **B** Total All-Cause Medical Costs, Stratified into Hospitalization, Outpatient, and Emergency Room Costs among VTE Patients in the Rivaroxaban versus Warfarin Cohorts

Patients who received rivaroxaban incurred significantly lower total all-cause medical costs than those who received warfarin (mean:$27,123 PPY vs. $29,637 PPY, cost difference(95%CI) = − $2515[− $4761;− $348]; Fig. 3B), which offset the higher pharmacy costs associated with rivaroxaban (mean:$7012 PPY vs. $5760 PPY, cost difference[95%CI] = $1252[$746;$1806], all p < 0.05; Fig. 3A and Table S4). The difference in total medical costs was mostly driven by lower outpatient costs (mean:$12,574 PPY vs. $14,224 PPY, cost difference[95% CI] = − $1650[− $2597;− $726]), particularly outpatient hospital costs (mean:$7560 PPY vs. $8722 PPY, cost difference[95%CI] = − $1162[− $1900;− $470], all p < 0.01; Fig. 3B and Table S4). Similar trends were observed for VTE-related healthcare costs (Table S4).

## Discussion

In this retrospective study based on health insurance claims data, rivaroxaban was associated with a statistically significant reduction in VTE recurrence relative to warfarin among VTE patients with obesity. Likewise, the rates of major bleeding were similar between both cohorts. Furthermore, rivaroxaban was associated with statistically significant lower all-cause medical costs and a nonsignificant numerically lower all-cause total healthcare costs vs. warfarin.

The results of this study build on the existing literature. In the EINSTEIN-DVT trial (rivaroxaban vs. enoxaparin/VKA for DVT), patients were stratified based on weight rather than BMI (the highest weight category was > 90 kg), and the number of VTE recurrences was low in both arms among patients > 90 kg (rivaroxaban:11/491, enoxaparin/VKA:11/486) [20]. A similar weight stratification was used in the EINSTEIN-PE trial (rivaroxaban vs. enoxaparin/VKA for PE); likewise, the number of recurrences was low in patients > 90 kg (rivaroxaban:13/683, enoxaparin/VKA:10/672) [21]. Therefore, despite the wealth of data provided by the EINSTEIN trials, the relative safety and efficacy of rivaroxaban versus warfarin remains uncertain in the obese population.

Several observational studies subsequently evaluated the safety and effectiveness of rivaroxaban in patients with morbid obesity [7–9, 13]. However, to the best of our knowledge, only Costa et al. addressed this research question in the broader population of patients with obesity (i.e., BMI ≥ 30 kg/m^2^) rather than patients with morbid obesity (i.e., BMI ≥ 40 kg/m^2^) [13]. Consistent with the current study, the authors found that the rates of VTE recurrence were significantly lower in rivaroxaban-treated patients than warfarin-treated patients and that the rates of major bleeding were similar [13]. Interestingly, the effect size observed for VTE recurrence at 12 months was larger in the study by Costa et al. [HR(95%CI) = 0.63(0.54;0.74)] than the current study [HR (95%CI) = 0.85(0.75;0.97)] [13]. This might be driven by the severity of VTE events in the two studies; in the present study, 49.5–50.8% of patients experienced a PE (with or without DVT) whereas this proportion was only 20.7–24.4% in the Costa et al. study [13].

The risk of VTE recurrence has been shown to increase linearly with BMI, with each 1-unit increase in BMI associated with a 4.4% higher risk of VTE recurrence [22]. Although the present study focused on a more inclusive population of VTE patients with obesity (rather than VTE patients with morbid obesity), the risk of VTE observed (~ 7–9% at 12 months) was within the range observed in previous studies of patients with morbid obesity (~ 1–17%) [6–9], but inconsistent length of follow-up and differences in patient characteristics limit comparisons across studies. Notwithstanding this limitation, this suggests that the risk of recurrent VTE may remain substantial in the broader population of VTE patients with obesity. Taken together, the results of the current study are consistent with the growing body of literature which suggests that rivaroxaban is a safe and effective option to reduce the risk of VTE recurrence in this population [23].

In the current study, patients initiated on rivaroxaban incurred significantly lower medical costs than those initiated on warfarin, which offset the higher pharmacy costs associated with rivaroxaban. The major driver of the difference in medical costs were outpatient costs, which may be lower among rivaroxaban users due to DOACs not requiring international normalized ratio monitoring [24]. Likewise, this may also explain the large difference in outpatient visits, which were more than two times less frequent among rivaroxaban users compared with warfarin users. These data suggest that rivaroxaban may be a cost-neutral alternative to VKAs among VTE patients with obesity.

### Limitations

The present study should be interpreted considering some limitations inherent to the retrospective nature of the analysis. First, height and weight data are not available in health insurance claims; thus, obesity was identified using ICD-9-CM and ICD-10-CM codes for high BMI rather than actual BMI values. Because of this, some VTE patients with obesity may not have been captured. However, research has shown that patients with codes for obesity are likely to be obese (i.e., high positive predictive value and high specificity) [25–28]. Second, VTE recurrences were defined based on diagnosis recorded during a hospitalization; therefore, recurrences in the outpatient setting were not captured. Third, mortality data were not available. Fourth, coding inaccuracies in administrative claims data may have led to the misidentification of some patients, although this limitation is expected to similarly impact both study cohorts. Similarly, the database may not contain information on all medications, particularly those administered in inpatient settings and over-the-counter medications (e.g., aspirin). Fifth, it was not possible to know whether all tablets supplied were actually taken by the patients. Sixth, while IPTW mitigated the risk of confounding due to observed variables, unmeasured confounders may have impacted results. Seventh, confounding by indication cannot be eliminated, because of the lack of randomization in an observational study. However, careful choice of study design and patient inclusion/exclusion criteria can help mitigate the potential risk of selection bias. Eighth, patients included were working age adults with commercial insurance; thus, results may not be generalized to other populations. Lastly, healthcare costs were assessed from the payer’s perspective and do not include indirect costs (e.g., productivity costs).

## Conclusion

In this retrospective cohort study, VTE patients with obesity that were initiated on rivaroxaban had a lower risk of VTE recurrence and a similar risk of major bleeding compared with those initiated on warfarin. The higher pharmacy costs associated with rivaroxaban were fully offset by reduced medical costs, resulting in similar total healthcare costs between rivaroxaban and warfarin users. Altogether, these data suggest that rivaroxaban is a safe, effective, and cost-neutral alternative to VKAs among VTE patients with obesity.

## Supplementary Information

Below is the link to the electronic supplementary material.

Supplementary file1 (DOCX 37 kb)
