# Supplementary material for: Effectiveness, safety, and healthcare costs associated with rivaroxaban versus warfarin among venous thromboembolism patients with obesity: a real-world study in the United States
Source: J Thromb Thrombolysis. 2022 May 13;54(3):438–48. doi: 10.1007/s11239-022-02661-1 (PMC9553828; doi:10.1007/s11239-022-02661-1)
Supplement: Supplementary file 1 — Supplementary file1 (DOCX 37 kb) [file 11239_2022_2661_MOESM1_ESM.docx]

**Supplemental Material**

**Figure S1.** Patient Disposition

AF: atrial fibrillation; BMI: body mass index; GPI: generic product identifier; ICD: international classification of disease; VTE: venous thromboembolism.

**Notes**:

1. A total of 122 rivaroxaban patients with >1 oral anticoagulant medications on the index date were excluded.

2. A total of 133 warfarin patients with >1 oral anticoagulant medications on the index date were excluded.

3. Continuous eligibility was defined as continuous health plan enrollment with medical and pharmacy coverage.

4. Baseline period was defined as the 12 months prior to the index date.

5. Recurrent VTE is defined as a hospitalization with a primary diagnosis of VTE.

**Table S1.** ICD-9-CM Codes for VTE Diagnosis

| **ICD-9-CM Code** | **Full Description** |
| --- | --- |
| **PE** | |
| 415.1 | Pulmonary embolism and infarction |
| 415.11 | Iatrogenic pulmonary embolism and infarction |
| 415.12 | Septic pulmonary embolism |
| 415.13 | Saddle embolus of pulmonary artery |
| 415.19 | Other pulmonary embolism and infarction |
| **DVT** | |
| 451.1 | Phlebitis and thrombophlebitis of deep veins of lower extremities |
| 451.11 | Phlebitis and thrombophlebitis of femoral vein (deep) (superficial) |
| 451.19 | Phlebitis and thrombophlebitis of other deep vessels of lower extremities |
| 451.2 | Phlebitis and thrombophlebitis of lower extremities, unspecified |
| 453.4 | Acute venous embolism and thrombosis of deep vessels of lower extremity |
| 453.40 | Acute venous embolism and thrombosis of unspecified deep vessels of lower extremity |
| 453.41 | Acute venous embolism and thrombosis of deep vessels of proximal lower extremity |
| 453.42 | Acute venous embolism and thrombosis of deep vessels of distal lower extremity |
| 453.8 | Acute venous embolism and thrombosis of other specified veins |
| 453.81 | Acute venous embolism and thrombosis of superficial veins of upper extremity |
| 453.82 | Acute venous embolism and thrombosis of deep veins of upper extremity |
| 453.83 | Acute venous embolism and thrombosis of upper extremity, unspecified |
| 453.84 | Acute venous embolism and thrombosis of axillary veins |
| 453.85 | Acute venous embolism and thrombosis of subclavian veins |
| 453.86 | Acute venous embolism and thrombosis of internal jugular veins |
| 453.87 | Acute venous embolism and thrombosis of other thoracic veins |
| 453.89 | Acute venous embolism and thrombosis of other specified veins |
| 453.9 | Embolism and thrombosis of unspecified site |

**Abbreviations:** DVT = deep vein thrombosis; ICD-9-CM = International Classification of Diseases, 9th Revision, Clinical Modification; VTE = venous thromboembolism; PE = pulmonary embolism

**Table S2.** ICD-10-CM Codes for VTE Diagnosis

| **ICD-10-CM Code** | **Full Description** |
| --- | --- |
| **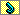PE** | |
| **I26** | **Pulmonary embolism** |
| I26.0 | Pulmonary embolism with acute cor pulmonale |
| I26.01 | Septic pulmonary embolism with acute cor pulmonale |
| I26.02 | Saddle embolus of pulmonary artery with acute cor pulmonale |
| I26.09 | Other pulmonary embolism with acute cor pulmonale |
| I26.9 | Pulmonary embolism without acute cor pulmonale |
| I26.90 | Septic pulmonary embolism without acute cor pulmonale |
| I26.92 | Saddle embolus of pulmonary artery without acute cor pulmonale |
| I26.99 | Other pulmonary embolism without acute cor pulmonale |
| **DVT** | |
| **I80** | **Phlebitis and thrombophlebitis** |
| I80.1 | Phlebitis and thrombophlebitis of femoral vein |
| I80.10 | Phlebitis and thrombophlebitis of unspecified femoral vein |
| I80.11 | Phlebitis and thrombophlebitis of right femoral vein |
| I80.12 | Phlebitis and thrombophlebitis of left femoral vein |
| I80.13 | Phlebitis and thrombophlebitis of femoral vein, bilateral |
| I80.2 | Phlebitis and thrombophlebitis of other and unspecified deep vessels of lower extremities |
| I80.20 | Phlebitis and thrombophlebitis of unspecified deep vessels of lower extremities |
| I80.201 | Phlebitis and thrombophlebitis of unspecified deep vessels of right lower extremity |
| I80.202 | Phlebitis and thrombophlebitis of unspecified deep vessels of left lower extremity |
| I80.203 | Phlebitis and thrombophlebitis of unspecified deep vessels of lower extremities, bilateral |
| I80.209 | Phlebitis and thrombophlebitis of unspecified deep vessels of unspecified lower extremity |
| I80.21 | Phlebitis and thrombophlebitis of iliac vein |
| I80.211 | Phlebitis and thrombophlebitis of right iliac vein |
| I80.212 | Phlebitis and thrombophlebitis of left iliac vein |
| I80.213 | Phlebitis and thrombophlebitis of iliac vein, bilateral |
| I80.219 | Phlebitis and thrombophlebitis of unspecified iliac vein |
| I80.22 | Phlebitis and thrombophlebitis of popliteal vein |
| I80.221 | Phlebitis and thrombophlebitis of right popliteal vein |
| I80.222 | Phlebitis and thrombophlebitis of left popliteal vein |
| I80.223 | Phlebitis and thrombophlebitis of popliteal vein, bilateral |
| I80.229 | Phlebitis and thrombophlebitis of unspecified popliteal vein |
| I80.23 | Phlebitis and thrombophlebitis of tibial vein |
| I80.231 | Phlebitis and thrombophlebitis of right tibial vein |
| I80.232 | Phlebitis and thrombophlebitis of left tibial vein |
| I80.233 | Phlebitis and thrombophlebitis of tibial vein, bilateral |
| I80.239 | Phlebitis and thrombophlebitis of unspecified tibial vein |
| I80.29 | Phlebitis and thrombophlebitis of other deep vessels of lower extremities |
| I80.291 | Phlebitis and thrombophlebitis of other deep vessels of right lower extremity |
| I80.292 | Phlebitis and thrombophlebitis of other deep vessels of left lower extremity |
| I80.293 | Phlebitis and thrombophlebitis of other deep vessels of lower extremity, bilateral |
| I80.299 | Phlebitis and thrombophlebitis of other deep vessels of unspecified lower extremity |
| I80.3 | Phlebitis and thrombophlebitis of lower extremities, unspecified |
| **I82** | **Other venous embolism and thrombosis** |
| I82.4 | Acute embolism and thrombosis of deep veins of lower extremity |
| I82.40 | Acute embolism and thrombosis of unspecified deep veins of lower extremity |
| I82.401 | Acute embolism and thrombosis of unspecified deep veins of right lower extremity |
| I82.402 | Acute embolism and thrombosis of unspecified deep veins of left lower extremity |
| I82.403 | Acute embolism and thrombosis of unspecified deep veins of lower extremity, bilateral |
| I82.409 | Acute embolism and thrombosis of unspecified deep veins of unspecified lower extremity |
| I82.41 | Acute embolism and thrombosis of femoral vein |
| I82.411 | Acute embolism and thrombosis of right femoral vein |
| I82.412 | Acute embolism and thrombosis of left femoral vein |
| I82.413 | Acute embolism and thrombosis of femoral vein, bilateral |
| I82.419 | Acute embolism and thrombosis of unspecified femoral vein |
| I82.42 | Acute embolism and thrombosis of iliac vein |
| I82.421 | Acute embolism and thrombosis of right iliac vein |
| I82.422 | Acute embolism and thrombosis of left iliac vein |
| I82.423 | Acute embolism and thrombosis of iliac vein, bilateral |
| I82.429 | Acute embolism and thrombosis of unspecified iliac vein |
| I82.43 | Acute embolism and thrombosis of popliteal vein |
| I82.431 | Acute embolism and thrombosis of right popliteal vein |
| I82.432 | Acute embolism and thrombosis of left popliteal vein |
| I82.433 | Acute embolism and thrombosis of popliteal vein, bilateral |
| I82.439 | Acute embolism and thrombosis of unspecified popliteal vein |
| I82.44 | Acute embolism and thrombosis of tibial vein |
| I82.441 | Acute embolism and thrombosis of right tibial vein |
| I82.442 | Acute embolism and thrombosis of left tibial vein |
| I82.443 | Acute embolism and thrombosis of tibial vein, bilateral |
| I82.449 | Acute embolism and thrombosis of unspecified tibial vein |
| I82.49 | Acute embolism and thrombosis of other specified deep vein of lower extremity |
| I82.491 | Acute embolism and thrombosis of other specified deep vein of right lower extremity |
| I82.492 | Acute embolism and thrombosis of other specified deep vein of left lower extremity |
| I82.493 | Acute embolism and thrombosis of other specified deep vein of lower extremity, bilateral |
| I82.499 | Acute embolism and thrombosis of other specified deep vein of unspecified lower extremity |
| I82.4Y | Acute embolism and thrombosis of unspecified deep veins of proximal lower extremity |
| I82.4Y1 | Acute embolism and thrombosis of unspecified deep veins of right proximal lower extremity |
| I82.4Y2 | Acute embolism and thrombosis of unspecified deep veins of left proximal lower extremity |
| I82.4Y3 | Acute embolism and thrombosis of unspecified deep veins of proximal lower extremity, bilateral |
| I82.4Y9 | Acute embolism and thrombosis of unspecified deep veins of unspecified proximal lower extremity |
| I82.4Z | Acute embolism and thrombosis of unspecified deep veins of distal lower extremity |
| I82.4Z1 | Acute embolism and thrombosis of unspecified deep veins of right distal lower extremity |
| I82.4Z2 | Acute embolism and thrombosis of unspecified deep veins of left distal lower extremity |
| I82.4Z3 | Acute embolism and thrombosis of unspecified deep veins of distal lower extremity, bilateral |
| I82.4Z9 | Acute embolism and thrombosis of unspecified deep veins of unspecified distal lower extremity |
| I82.6 | Acute embolism and thrombosis of veins of upper extremity |
| I82.60 | Acute embolism and thrombosis of unspecified veins of upper extremity |
| I82.601 | Acute embolism and thrombosis of unspecified veins of right upper extremity |
| I82.602 | Acute embolism and thrombosis of unspecified veins of left upper extremity |
| I82.603 | Acute embolism and thrombosis of unspecified veins of upper extremity, bilateral |
| I82.609 | Acute embolism and thrombosis of unspecified veins of unspecified upper extremity |
| I82.61 | Acute embolism and thrombosis of superficial veins of upper extremity |
| I82.611 | Acute embolism and thrombosis of superficial veins of right upper extremity |
| I82.612 | Acute embolism and thrombosis of superficial veins of left upper extremity |
| I82.613 | Acute embolism and thrombosis of superficial veins of upper extremity, bilateral |
| I82.619 | Acute embolism and thrombosis of superficial veins of unspecified upper extremity |
| I82.62 | Acute embolism and thrombosis of deep veins of upper extremity |
| I82.621 | Acute embolism and thrombosis of deep veins of right upper extremity |
| I82.622 | Acute embolism and thrombosis of deep veins of left upper extremity |
| I82.623 | Acute embolism and thrombosis of deep veins of upper extremity, bilateral |
| I82.629 | Acute embolism and thrombosis of deep veins of unspecified upper extremity |
| I82.A | Embolism and thrombosis of axillary vein |
| I82.A1 | Acute embolism and thrombosis of axillary vein |
| I82.A11 | Acute embolism and thrombosis of right axillary vein |
| I82.A12 | Acute embolism and thrombosis of left axillary vein |
| I82.A13 | Acute embolism and thrombosis of axillary vein, bilateral |
| I82.A19 | Acute embolism and thrombosis of unspecified axillary vein |
| I82.B | Embolism and thrombosis of subclavian vein |
| I82.B1 | Acute embolism and thrombosis of subclavian vein |
| I82.B11 | Acute embolism and thrombosis of right subclavian vein |
| I82.B12 | Acute embolism and thrombosis of left subclavian vein |
| I82.B13 | Acute embolism and thrombosis of subclavian vein, bilateral |
| I82.B19 | Acute embolism and thrombosis of unspecified subclavian vein |
| I82.C | Embolism and thrombosis of internal jugular vein |
| I82.C1 | Acute embolism and thrombosis of internal jugular vein |
| I82.C11 | Acute embolism and thrombosis of right internal jugular vein |
| I82.C12 | Acute embolism and thrombosis of left internal jugular vein |
| I82.C13 | Acute embolism and thrombosis of internal jugular vein, bilateral |
| I82.C19 | Acute embolism and thrombosis of unspecified internal jugular vein |
| I82.90 | Acute embolism and thrombosis of unspecified vein |

ICD-10-CM: International Classification of Diseases, 10th Revision, Clinical Modification; VTE: venous thromboembolism; PE: pulmonary embolism; DVT: deep vein thrombosis; ICD: International Classification of Diseases

**Table S3.** BMI-Related Diagnosis Codes to Identify Obesity

| **ICD Code** | **Description** | **Category^1^** |
| --- | --- | --- |
| **ICD-9-CM** |  |  |
| 278.00 | Obesity, unspecified | Obesity |
| 278.01 | Morbid obesity | Morbid Obesity |
| 278.03 | Obesity hypoventilation syndrome | Obesity |
| V85.30 | Body Mass Index 30.0-30.9, adult | Obesity |
| V85.31 | Body Mass Index 31.0-31.9, adult | Obesity |
| V85.32 | Body Mass Index 32.0-32.9, adult | Obesity |
| V85.33 | Body Mass Index 33.0-33.9, adult | Obesity |
| V85.34 | Body Mass Index 34.0-34.9, adult | Obesity |
| V85.35 | Body Mass Index 35.0-35.9, adult | Obesity |
| V85.36 | Body Mass Index 36.0-36.9, adult | Obesity |
| V85.37 | Body Mass Index 37.0-37.9, adult | Obesity |
| V85.38 | Body Mass Index 38.0-38.9, adult | Obesity |
| V85.39 | Body Mass Index 39.0-39.9, adult | Obesity |
| V85.41 | Body Mass Index 40.0-44.9, adult | Morbid Obesity |
| V85.42 | Body Mass Index 45.0-49.9, adult | Morbid Obesity |
| V85.43 | Body Mass Index 50.0-59.9, adult | Morbid Obesity |
| V85.44 | Body Mass Index 60.0-69.9, adult | Morbid Obesity |
| V85.45 | Body Mass Index 70 and over, adult | Morbid Obesity |
| **ICD-10-CM** |  |  |
| E66.0 | Obesity due to excess calories | Obesity |
| E66.01 | Morbid (severe) obesity due to excess calories | Morbid Obesity |
| E66.09 | Other obesity due to excess calories | Obesity |
| E66.1 | Drug-induced obesity | Obesity |
| E66.2 | Morbid (severe) obesity with alveolar hypoventilation | Morbid Obesity |
| E66.8 | Other obesity | Obesity |
| E66.9 | Obesity, unspecified | Obesity |
| Z68.3 | Body mass index (BMI) 30-39, adult | Obesity |
| Z68.30 | Body mass index (BMI) 30.0-30.9, adult | Obesity |
| Z68.31 | Body mass index (BMI) 31.0-31.9, adult | Obesity |
| Z68.32 | Body mass index (BMI) 32.0-32.9, adult | Obesity |
| Z68.33 | Body mass index (BMI) 33.0-33.9, adult | Obesity |
| Z68.34 | Body mass index (BMI) 34.0-34.9, adult | Obesity |
| Z68.35 | Body mass index (BMI) 35.0-35.9, adult | Obesity |
| Z68.36 | Body mass index (BMI) 36.0-36.9, adult | Obesity |
| Z68.37 | Body mass index (BMI) 37.0-37.9, adult | Obesity |
| Z68.38 | Body mass index (BMI) 38.0-38.9, adult | Obesity |
| Z68.39 | Body mass index (BMI) 39.0-39.9, adult | Obesity |
| Z68.4 | Body mass index (BMI) 40 or greater, adult | Morbid Obesity |
| Z68.41 | Body mass index (BMI) 40.0-44.9, adult | Morbid Obesity |
| Z68.42 | Body mass index (BMI) 45.0-49.9, adult | Morbid Obesity |
| Z68.43 | Body mass index (BMI) 50-59.9 , adult | Morbid Obesity |
| Z68.44 | Body mass index (BMI) 60.0-69.9, adult | Morbid Obesity |
| Z68.45 | Body mass index (BMI) 70 or greater, adult | Morbid Obesity |

**Abbreviations:** BMI = body mass index; ICD-9-CM = International Classification of Diseases, 9th Revision, Clinical Modification; ICD-10-CM = International Classification of Diseases, 10th Revision, Clinical Modification.

**Note:**

1. Obesity was defined as BMI ≥30 kg/m^2^, and morbid obesity was defined as BMI ≥40 kg/m^2^

**Table S4.** Healthcare Costs among Rivaroxaban vs. Warfarin Cohorts – Intention-to-Treat

| **Healthcare costs** | **Rivaroxaban** | **Warfarin** | **Cost difference [A]-[B] (95% CI)^1^** | **P-value^1^** |
| --- | --- | --- | --- | --- |
|  | **[A]** | **[B]** |  |  |
|  | **N = 8,666** | **N = 5,946** |  |  |
| **Observation period,^2^ months, mean ± SD [median]** | 9.7 ± 3.8 [12] | 9.6 ± 3.9 [12] |  |  |
| **Healthcare costs,^3^ $US 2019 PPY, mean ± SD** |  |  |  |  |
| *All-cause* |  |  |  |  |
| Total healthcare costs | $34,135 ± 80,326 | $35,398 ± 92,395 | -1,263 (-3,571, 1,052) | 0.277 |
| Total medical costs | $27,123 ± 78,099 | $29,637 ± 87,407 | -2,515 (-4,761, -348) | 0.020 |
| Hospitalization costs | $12,372 ± 67,824 | $13,087 ± 75,703 | -714 (-2,581, 1,056) | 0.445 |
| ER costs | $2,176 ± 7,543 | $2,326 ± 7,277 | -150 (-391, 76) | 0.188 |
| OP costs | $12,574 ± 26,839 | $14,224 ± 31,881 | -1,650 (-2,597, -726) | <0.001 |
| OP hospital visit costs | $7,560 ± 22,017 | $8,722 ± 24,749 | -1,162 (-1,900, -470) | 0.004 |
| Office visit costs | $2,265 ± 4,291 | $2,396 ± 4,242 | -131 (-290, 14) | 0.068 |
| Other visit costs | $2,749 ± 10,456 | $3,106 ± 14,343 | -357 (-819, 41) | 0.088 |
| Pharmacy costs | $7,012 ± 11,600 | $5,760 ± 22,805 | 1,252 (746, 1,806) | <0.001 |
| *VTE-related^4^* |  |  |  |  |
| Total medical costs | $8,777 ± 55,574 | $9,420 ± 64,500 | -643 (-2,039, 760) | 0.373 |
| Hospitalization costs | $6,110 ± 54,578 | $5,992 ± 63,316 | 118 (-1,211, 1,468) | 0.890 |
| ER costs | $441 ± 3,242 | $497 ± 3,216 | -56 (-139, 21) | 0.156 |
| OP costs | $2,225 ± 7,230 | $2,930 ± 8,215 | -705 (-936, -469) | <0.001 |
| OP hospital visit costs | $1,509 ± 6,806 | $1,947 ± 7,798 | -438 (-655, -217) | <0.001 |
| Office visit costs | $499 ± 1,177 | $678 ± 1,417 | -180 (-224, -132) | <0.001 |
| Other visit costs | $217 ± 1,392 | $305 ± 1,469 | -88 (-145, -22) | 0.004 |

**Abbreviations:** CI: confidence interval; ER: emergency room; OP: outpatient; PPY: per patient-years; SD: standard deviation; VTE: venous thromboembolism

**Notes:**

1. Confidence intervals and p-values were calculated using non-parametric bootstrap procedure (B=499).
2. The observation period spans from the index date until the earliest date between 12 months, health plan disenrollment, end of data availability, or presence of both diagnosis and treatment of cancer (at the later of the two dates).
3. All costs were inflation-adjusted to 2019 US dollars based on the medical care component of the Consumer Price Index.
4. Healthcare costs are considered VTE-related if it is associated with a primary or secondary diagnosis (i.e., identified in any other diagnosis fields) of venous thromboembolism.
